# Advancements in Surgical Management of Megaureters

**DOI:** 10.1007/s11934-024-01214-8

**Published:** 2024-07-02

**Authors:** Timothy C. Boswell

**Affiliations:** Department of Urology, Children’s of Alabama and University of Alabama at Birmingham, 1600 7th Avenue South, Lowder Suite 318, Birmingham, AL 35233 USA

**Keywords:** Megaureter, Vesicoureteral Reflux, Primary Obstructive Megaureter, Ureteral Balloon Dilation, Minimally Invasive Surgery, Robotic Surgery

## Abstract

**Purpose of Review:**

To review and describe the recent evolution of surgery for the various types of pediatric megaureter.

**Recent Findings:**

Megaureter management first relies on determining the underlying cause, whether by obstruction, reflux, or a combination, and then setting appropriate surgical indications because many cases do not require surgery as shown by observation studies. Endoscopic balloon dilation has been on the rise as a major treatment option for obstructive megaureter, while refluxing megaureters can also be treated by laparoscopic and robotic techniques, whether extravesically or transvesicoscopically. During ureteral reimplantation, tapering is sometimes necessary to address the enlarged ureter, but there are also considerations for not tapering or for tapering alternatives.

**Summary:**

Endoscopic and minimally invasive surgeries for megaureter have been the predominant focus of recent megaureter literature. These techniques still need collaborative prospective studies to better define which surgeries are best for patients needing megaureter interventions.

## Introduction

Megaureter is defined as an enlargement of the ureter, usually greater than 7 or 8 mm, and typically identified by ultrasound in pediatric patients [1–3]. Megaureter is an imaging finding only, and the underlying cause must be elucidated to determine appropriate management options, whether medical or surgical. The classic breakdown of etiologies is King’s classification which is a two by two grid describing the presence or absence each of vesicoureteral reflux (VUR) and obstruction [4]. This classification gives the two main categories of primary obstructed (non-refluxing) megaureter (POM) and refluxing (non-obstructed) megaureter (dilating VUR), as well as the two less common groups of obstructed refluxing megaureter (ORM) and non-obstructive non-refluxing megaureter. Management in most scenarios is close observation without surgery; however, the decision for surgery as well as the surgical techniques have evolved over time. Each of these pathologies has unique aspects in management, but the general aspects of surgical management for a megaureter involve relieving obstruction or correcting reflux without introducing either in the post-surgical anatomy. Surgical principles are underscored by normal ureteral form and function, whereby antegrade flow of urine is permitted by an adequate ureteral diameter, allowing opposing wall coaptation for effective peristalsis [5]. Likewise, reflux is prevented by an adequate intramural submucosal tunnel to create an effective valve mechanism [6], combined with an appropriate ureteral orifice morphology [7, 8]. In treating pathologic versions of megaureter, the themes across pediatric urology of decreasing invasiveness by pursuing endoscopic and/or laparoscopic and robotic surgical options have likewise applied, as we have seen with endoscopic balloon dilation as well as laparoscopic and robotic versions of the highly successful traditional open ureteroneocystostomy (UNC) techniques.

## Determining Megaureter Type, and Indication for Surgery – Critical Underpinnings

Determining the specific megaureter type and defining the criteria for escalating to operative management are both critical for clinical practice, and likewise essential for appreciating the literature on the evolution of megaureter management. To this end, the typical evaluation of megaureter begins with a voiding cystourethrogram (VCUG) which, if showing no reflux, strongly suggests an obstructive process particularly in the presence of ultrasound findings of significant ureteral dilation, tortuosity, and/or intraluminal debris. Elucidating the presence of an obstructive element is the paramount priority, as obstruction (more so than reflux) has a higher morbidity rate and necessitates aspects of the surgical treatment (when necessary), such as requiring excision of the obstructive segment during reimplantation. The ultrasound and VCUG also provide evidence to rule out a secondary megaureter (such as from posterior urethral valves, neurogenic bladder, etc.), as confirming primary megaureter is critical as well before pursuing megaureter treatments. The typical next test to assess for obstruction is a diuretic renogram (DR) which can define split renal function and assess renal and ureteral washout for evidence of obstruction. Even when obstruction is confirmed, giving the diagnosis of POM, the recommended management is observation given that the majority of cases (around 72%) will improve and even resolve over time [9, 10]. When megaureter resolves, it occurs with gradual dilatation improvement from proximal to distal [11]. Factors associated with lower likelihood of resolution are higher grades of hydronephrosis and larger ureteral diameter [12], with one study reporting Society for Fetal Urology (SFU) hydronephrosis grades 3–4 and ureteral diameter > 13 mm as significant predictors for meeting surgical criteria [13]. A more recent prospective study of 50 ureters used a ureteral diameter of 10 mm as a cutoff, and found a 76% resolution rate over median 5 years for those less than 10 mm, and those ≥ 10 mm had a 17% resolution rate over a median of 9 years [14].

Surgical indications in POM studies most commonly cite the British Association of Pediatric Urologists 2014 consensus statement guidelines [3]. While these allude to clinical criteria such as urinary tract infections (UTI), pain, and nephrolithiasis, as well as progressive dilation on ultrasound, they focus their discussion on DR findings that should prompt surgery. Specifically, a delayed T ½ (greater than 20 min) or delayed washout curve is not enough to prompt surgery in an asymptomatic patient with stable or improving dilation. Rather, DR findings of an initial differential renal function (DRF) of < 40% or a drop in DRF by 5% or more on serial scans indicate surgery due to loss of function. Utilizing delayed washout as a surgical trigger is not recommended because in studies on both ureteropelvic junction obstruction and ureterovesical junction obstruction, kidneys with obstruction by delayed washout have continued to grow normally, maintain function, and even resolve their dilation. Likewise, washout curves in megaureter are particularly prone to inaccuracy at baseline, given that the region of interest outlined must include a dilated and often tortuous ureter, which may have various degrees of filling after diuretic administration and before draining [15, 16]. Given the limitations of renography in a megaureter, as well as the radiation exposure, cost, intravenous access, and bladder catheterization, some providers opt for no (or less frequent) renography and rather rely predominantly on serial ultrasound findings showing significantly increasing ureteral and pyelocaliceal dilation as an indication for surgery, in addition to clinical reasons above. These facts are particularly salient when considering the surgical outcomes literature, given that studies may utilize different surgical indication criteria as a limitation for comparing outcomes across studies.

The next section will continue with the focus on primary obstructive megaureter, given that moving on to clinical treatment of a refluxing megaureter can only be done if obstruction has first been ruled out. This brings up an important, and perhaps under-recognized, point about the existence of obstructed refluxing megaureter (ORM). Put simply, vesicoureteral reflux on a VCUG does not rule out obstruction. Findings on a VCUG that suggest an obstructive component in addition to reflux include the appearance of a normal or narrow caliber ureter at its distal 1–2 cm with significant ureterectasis proximal, as well as initial diluted contrast filling of a dilated ureter, or poor drainage of contrast from the ureter into the bladder on a late film [17]. Importantly, the main place in the literature where ORM is described is in cases of obstruction after endoscopic treatment of VUR with dextranomer/hyaluronic acid [18–20]. This fact should serve as a reminder to those considering treating cases of high-grade VUR with endoscopic injection, which has been done in significant volume in recent years [21], to first consider if an obstructive element could be at play.

## The Evolution of Surgery for Primary Obstructive Megaureter

The historical gold standard for surgical treatment of POM has been ureteral reimplantation (UNC) with or without ureteral tapering. In infant cases, when reimplanting a dilated ureter into a small bladder can be a challenge (or, when needing immediate temporizing drainage in the setting of active infection), a cutaneous ureterostomy is created with plans for subsequent UNC. Typically, tapering is not required if months of drainage via the ureterostomy has allowed for downsizing of the megaureter. Several additional strategies have developed in efforts to minimize the need for repeat open surgeries or tapering, while aiming to equal or improve the success seen with UNC. Ureteral stent placement is an option for internal drainage which can give more time for spontaneous resolution of megaureter, or can bridge infant patients until older to permit simpler reimplantation. Studies mostly from the 2000s reported that ureteral stents for POM were reasonably effective, in that about half of patients did not require subsequent surgery after 3 to 6 months of stenting. However, the fallbacks were that many infant POM ureteral stents required open placement, or had problems with UTI, stone formation, or migration [22–24]. More recently, a larger study (including 29 patients and 35 ureters) with longer follow-up (6 years) showed that 25% did not require subsequent surgery and there was a 40% rate of issues during the stenting period including UTI, hematuria, stones, and stent migration [25]. Other options arose which effectively sought to trade obstruction for reflux. These include an upfront dismembered refluxing reimplant, popularized by Kaefer, working as an internal temporary diversion rather than diverting to the skin via ureterostomy. In the report on 19 ureters managed this way, 18 went on to definitive surgery (as planned) after 1 year old: two had nephrectomy, and of the 16 undergoing reimplantation, 13 still required ureteral tapering [26, 27]. An evolution of this technique was proposed wherein a non-dismembered side-to-side refluxing reimplant is performed [28]. The group reporting this procedure proposed that the refluxing side-to-side technique could be considered the final management (with no follow-up surgery), particularly in circumcised males if there were no UTIs in the follow-up period.

Endoscopic balloon dilation (EBD) has gained popularity in the past 20 years for the treatment of POM, as it represents a completely endoscopic procedure that can typically eliminate the obstruction without introducing de novo VUR. For EBD, a high-pressure balloon is inflated under cystoscopic and fluoroscopic guidance to dilate the distal ureter and ureterovesical junction [29–47]. A cutting balloon or laser can also be employed in stenoses refractory to dilation alone [31, 37, 46, 48–50]. This procedure was first described in 1998 in Spain [29] with early small series from multiple centers up to the early 2010s. Most studies came out of Europe, predominantly Spain, Italy, and France except for a few series from other continents including only one from the United States (U.S.) [31], until recently [47]. Over the last decade, the number of studies and cases has risen significantly with later series involving larger numbers including 40 + [41, 45, 46] and even 100 + ureters [42].

Systematic reviews [51–54] of these series have borne out the primary advantage of EBD which is completely endoscopic treatment that is frequently a definitive management, not requiring subsequent ureteral reimplantation. Success rates, defined variably by lack of need for traditional surgery or improvement on imaging, range from 60 to 100% in the published studies. The theoretical downside of dilating the UVJ would be the introduction of VUR. However, Garcia-Aparicio et al. showed a 27% rate of postoperative VUR after dilating to 18 or 21 Fr by performing systematic VCUGs in follow-up [38]. Most study protocols only called for a postoperative VCUG in the setting of a febrile UTI during the follow-up period, and in this setting an approximately 8% rate of clinically significant VUR has been detected so far [53]. Challenges with EBD can be encountered with intubating the stenotic orifice or navigating stent placement into a tortuous ureteral system, but most studies describe technical feasibility on the first attempt and, rarely, repeat dilation or pre-stenting for a period can permit completion. Later studies have even described dilation without stent placement [45] and without fluoroscopy [42], but most describe fluoroscopic guidance and placement of a single stent. Although, the two U.S. studies in the literature describe the use of tandem stents [31, 47]. That there are only two U.S. studies on this topic in the literature to date would suggest low adoption so far in North America, but the rising description worldwide of EBD would suggest growing implementation related to advantages compared to the prior treatment options described above.

The primary caveat to EBD, as defined by a quality analysis from the European Association of Urology and European Society for Pediatric Urology (EAU/ESPU) systematic review [53], is that nearly all studies supporting EBD are retrospective single-center series, representing a relatively low level of evidence. This fact would suggest the need for a prospective study in the pediatric urology community, and perhaps one comparing key outcomes to the above-described alternatives would be a worthwhile next venture in the modern surgical management of primary obstructive megaureter.

## Open Surgical Principles Applied Laparoscopically and Robotically

In some patients, surgical reconstruction is necessary (or preferred) over endoscopic treatment. The other clear trend in recent years has been to pursue the use of minimally invasive surgery (MIS) techniques to accomplish the equivalent of traditional open UNC. Generally, this has been done by either extravesical Lich-Gregoir reimplant laparoscopically, or intravesical reimplant varieties (Cohen cross-trigonal, Glenn-Anderson advancement, etc.) which require a transvesicoscopic approach. A systematic review of studies comparing laparoscopic extravesical and transvesicoscopic techniques suggests that skilled operators achieve comparable success rates between the two approaches [55]. These (particularly transvesicoscopic), however, are performed by select surgeons at select centers given the challenges compared to open surgery: for example, the largest transvesicoscopic series from a single surgeon included 182 patients, 317 ureters, and all patients were selected to be 3 years or older with less than grade 5 VUR to prevent the need for tapering [56]. Most other transvesicoscopic series include significantly lower numbers [57–62]. Laparoscopic techniques like this have not had broad uptake related to technical challenges and concern by some that the advantages may be marginal compared to open surgery. Additionally, the cosmesis and recovery from a small Pfannenstiel incision may be difficult to improve upon, even with laparoscopy.

Robotic-assisted laparoscopic approaches overcome some of the technical challenges (of laparoscopic suturing, in particular) and as such have the potential for broader uptake in the setting of appropriate resources. While initial series on robotic ureteral reimplantation for primary VUR showed favorable outcomes [63, 64], there was a general hesitation when later series suggested lower efficacy and higher complication rates compared to open techniques [65–67]. More recently, series have shown improvement in robotic success rates [68, 69], perhaps illustrating the general learning curve associated with progress. Updated systematic reviews comparing open and robotic techniques for VUR have shown similar overall efficacy, possibly shorter hospital stay with the robotic approach (although this may be driven by practice pattern), but also typically longer robotic operative time [70–72]. The experience with laparoscopic and robotic reimplantation has grown to the point that the recently updated EAU/ESPU guideline on VUR acknowledges robotic and laparoscopic extravesical and transvesicoscopic techniques as reasonable options in terms of resolution and complication rates [73]. These studies, again, represent surgical treatments for primary VUR, which often does not involve a significantly dilated ureter.

When considering megaureter specifically, there have been some MIS studies focusing on the dilated ureter. A recent multi-institutional European study group on VUR compared open and robotic ureteral reimplantation in children with high grade (grades 4 and 5) VUR. Nine centers retrospectively reviewed 135 cases with mean age 11 months old and found a 94% clinical success in the open group and 98.5% clinical success in the robotic group. Notably, these were all cases that did not require tapering. The robotic group showed improved recovery parameters including shorter indwelling catheter time and shorter hospital stay [74]. Generally, many authors describe that the benefits of the robotic approach stand out in older patients or those with complex anatomy [75, 76].

Laparoscopic and robotic techniques for POM have also been described. Most studies employ an extravesical Lich-Gregoir technique after dismembering the obstructed ureter, with or without tapering. The largest series reported on 18 patients with a range of ages from six months to 15 years old who underwent robotic Lich-Gregoir. Of these, 39% underwent tapering intracorporeally, and all patients had improved hydronephrosis without the need for subsequent surgery over median two year follow-up [77]. Another series of studies described a laparoscopic (and subsequently robotic) extravesical cross-trigonal technique whereby the POM was reimplanted extravesically across the posterior bladder to increase tunnel length compared to that available for a standard Lich-Gregoir [78–80]. When 47 laparoscopic and 48 robotic cases by these techniques were compared across four centers, the result was similar success rates (94–97% over 8–12 month median follow-up) and low high-grade complication rates (2–4%) [81]. Transvesicoscopic techniques have likewise been applied for POM, but can be challenging. Li et al. compared laparoscopic transvesicoscopic Cohen reimplants to laparoscopic single-site Lich-Gregoir reimplants and found similar success and complication rates, but longer operative time and hospitalization for the transvesicoscopic technique [82]. One potential simplification for extravesical laparoscopic management of a dilated and tortuous megaureter can be extracorporeal tapering as illustrated by this study and others [82–86]. Another group employed a robotic technique for 16 infants wherein they extended the Lich-Gregoir to the anterior bladder wall in order to accomplish a longer tunnel. Of these, 25% required tapering and they reported a 94% success rate [85]. In comparing robotic to open POM reimplant outcomes, one single-center series from Italy included 11 robotic cases and 12 open cases and reported a similar OR time, shorter hospital stay for robotic cases, as well as similar success (91–92%) and complication (8–9%) rates [87].

Overall, select surgeons and centers are pursuing and publishing on laparoscopic and robotic surgeries for refluxing and obstructed megaureters. The literature to date in toto alludes to the fact that there are significant learning curves with limited broad update. Time to proficiency may be even further extended if endoscopic treatment rates via injection for VUR and balloon dilation for POM continue to rise as suggested by increasing reports in the literature. Furthermore, while transvesicoscopic techniques are depicted in the literature, they are rather infrequent due to the challenges and are likely to remain less common until smaller and more advanced robotic platforms, such as a miniaturized single-port platform, become available.

## To Taper or Not to Taper

A unique aspect of megaureter reimplantation, more typically for an obstructive than a refluxing cause, is the potential need for ureteral tapering or tailoring. The pressure or impetus to taper in the modern era, however, is likely less pronounced than before for several reasons. First, the fundamental urological principle holds true that obstruction is worse than reflux. This fact is employed by the several surgical approaches for POM described above [26–28] which effectively trade obstruction for reflux as the lesser of two evils. Second, in the absence of infection, the field of pediatric urology has become gradually more permissive towards reflux in general. This has been evidenced by increasing observational management of VUR with decreasing surgical management over the past decades [88–90], not only waiting for reflux resolution but also observing in many instances as long as there are no significant clinical problems (i.e. febrile UTIs). Furthermore, many surgical outcome studies for POM and VUR no longer employ a planned VCUG during follow-up to define radiologic success, but rely solely on clinical success, wherein investigation with a VCUG is only pursued when prompted by a febrile UTI or other similar clinical problem. On the other side of the risk–benefit equation, UNC involving tapering has a higher operative complexity and higher complication rate, including urine leak, stricture, and VUR (whether by incompetent tunnel or ureterovesical fistula). Taking all these facts in the balance, the incentives in the current era point to a lower impetus to taper a megaureter when the necessity is in question.

For these reasons, ureteral tapering is likely done less frequently than in the past, although no study has borne out this suspicion. Important concepts associated with this logic, though, do appear in the literature that the surgeon should consider when undertaking megaureter surgery wherein the decision on tapering must be made. One concept is that a full 5:1 tunnel length to ureteral diameter ratio, as originally proposed by Paquin [6], may not be necessary [91]. This ratio in the megaureter can be challenging to achieve, especially in a small bladder. Permissiveness to a smaller ratio permits lesser (or no) tapering, a shorter tunnel, or both. Babu described what he called “mini reimplantation” of the megaureter wherein he employed no tapering during intravesical reimplant. He developed a 2:1 ratio tunnel in 13 patients wherein only two had postoperative reflux. This was a similar reflux outcome to 15 similar patients undergoing classic Cohen reimplants after excisional tapering, but the tapered cases had a higher rate of obstruction and higher overall need for reoperation [92]. Villanueva similarly presented a series of nine infants less than 6 months old requiring surgery for obstructive megaureter (4 ectopic, 5 POM). Instead of cutaneous ureterostomy, he performed a “mini” extravesical reimplant with a 2–3 cm tunnel regardless of ureteral diameter. He also performed “mini-tapering” in the last five patients wherein the distal 2–3 cm were tapered via the adventitia-sparing excisional technique described by Ossandon [93]. Two of the first four developed postoperative reflux, prompting his adaption to employ “mini- tapering” in the final cases, and none of the last five had clinically evident VUR over median follow-up of 44 months [94].

Further, it is not only the ratio of ureteral tunnel length to diameter that prevents reflux, but the configuration of the orifice may contribute to reflux prevention [7, 8]. Another valve mechanism addressing orifice configuration is the nipple valve [95–97]. This has recently been employed in the surgical management of POM, particularly in infant bladders that have limited domain for tunneling. Babu described laparoscopically securing the megaureter with 5 mm protruding into the bladder, combined with a short extravesical tunnel, as the treatment for 11 patients with POM ranging from five to 24 months old. He saw no VUR in this series on six month follow-up VCUGs [98]. Another group described a similar technique for POM, in which they employed no tapering and no tunnel, but instead secured the megaureter protruding into the bladder (with the distal end everted circumferentially) to achieve a ratio of intravesical length of nipple to ureteral diameter of about 2:1. In 13 cases, 11 were successful with one developing obstruction and one reflux. Several had cystoscopic follow-up and demonstrated persistence of a clear nipple-shaped orifice within the bladder [99]. In comparing tapered and non-tapered reimplants for POM, one series of 16 tapered ureters and 22 non-tapered ureters of similar initial diameter showed a higher rate of hydronephrosis resolution in the non-tapered group (50% vs. 19%), and each group needed one reoperation (one tapered for stricture, and one non-tapered for VUR) [100]. These studies illustrate the techniques and alternatives to tapering in the current era, which is more permissive of VUR than before.

## The Path Forward and Conclusions

Surgical treatment of the megaureter continues to grow and evolve. Of paramount importance is determining the presence or absence of obstruction, and maintaining the understanding that even when reflux is present that there can be an obstructive component as well. Proving obstruction still depends largely on diuretic renography, which has its qualms, and so there are certainly opportunities for improvement in the testing and definition for the presence or absence of obstruction. Modifications of the Whitaker test [101] and MR urography [102] have been attempted, but an ideal accurate and non-invasive test for ureteral obstruction still needs to be developed. Likewise, as has been alluded to above, the majority of the literature on surgical management of the megaureter comes from retrospective and single-center series. To further advance the field will require planning for prospective collaborative studies utilizing pre-defined surgical criteria and follow-up protocols. This will be particularly important for assessing newer technologies such as that seen employed increasingly with endoscopic balloon dilation for POM, as well as evaluations of future smaller and more sophisticated surgical tools and robotic platforms that are in development and will likely increasingly impact the field.

## Data Availability

No datasets were generated or analysed during the current study.

## References

[1] Cussen LJ. Dimensions of the normal ureter in infancy and childhood. Invest Urol. 1967;5(2):164–78. 6052953

[2] Hellström M, Hjälmås K, Jacobsson B, Jodal U, Odén A. Normal ureteral diameter in infancy and childhood. Acta Radiol Diagn (Stockh). 1985;26(4):433–9. 4050524 10.1177/028418518502600412

[3] Farrugia MK, Hitchcock R, Radford A, Burki T, Robb A, Murphy F. British Association of Paediatric Urologists consensus statement on the management of the primary obstructive megaureter. J Pediatr Urol. 2014;10(1):26–33. 24206785 10.1016/j.jpurol.2013.09.018

[4] King LR. Megaloureter: definition, diagnosis and management. J Urol. 1980;123(2):222–3. 7354523 10.1016/S0022-5347(17)55867-X

[5] Woodburne RT, Lapides J. The ureteral lumen during peristalsis. Am J Anat. 1972;133(3):255–8. 5026654 10.1002/aja.1001330302

[6] Paquin AJ. Ureterovesical anastomosis: the description and evaluation of a technique. J Urol. 1959;82:573–83. 14430329 10.1016/S0022-5347(17)65934-2

[7] Lyon RP, Marshall S, Tanagho EA. The ureteral orifice: its configuration and competency. J Urol. 1969;102(4):504–9. 5343494 10.1016/S0022-5347(17)62184-0

[8] Kirsch AJ, Kaye JD, Cerwinka WH, Watson JM, Elmore JM, Lyles RH, et al. Dynamic hydrodistention of the ureteral orifice: a novel grading system with high interobserver concordance and correlation with vesicoureteral reflux grade. J Urol. 2009;182(4S):1688–93. 19692002 10.1016/j.juro.2009.02.061

[9] Baskin LS, Zderic SA, Snyder HM, Duckett JW. Primary dilated megaureter: long-term followup. J Urol. 1994;152(2 Pt 2):618–21. 8021983 10.1016/S0022-5347(17)32665-4

[10] Di Renzo D, Aguiar L, Cascini V, Di Nicola M, McCarten KM, Ellsworth PI, et al. Long-term followup of primary nonrefluxing megaureter. J Urol. 2013;190(3):1021–6. 23500640 10.1016/j.juro.2013.03.008

[11] Randhawa H, Jones C, McGrath M, Braga LH. Non-refluxing primary megaureter in children resolves from proximal to distal. Urology. 2023;182:225–30. 37776954 10.1016/j.urology.2023.09.005

[12] McLellan DL, Retik AB, Bauer SB, Diamond DA, Atala A, Mandell J, et al. Rate and predictors of spontaneous resolution of prenatally diagnosed primary nonrefluxing megaureter. J Urol. 2002;168(5):2177–80; discussion 2180. 10.1016/S0022-5347(05)64348-0 12394754

[13] Chertin B, Pollack A, Koulikov D, Rabinowitz R, Shen O, Hain D, et al. Long-term follow up of antenatally diagnosed megaureters. J Pediatr Urol. 2008;4(3):188–91. 18631923 10.1016/j.jpurol.2007.11.013

[14] Ranawaka R, Hennayake S. Resolution of primary non-refluxing megaureter: an observational study. J Pediatr Surg. 2013;48(2):380–3. 23414869 10.1016/j.jpedsurg.2012.11.017

[15] Gordon I. Diuretic renography in infants with prenatal unilateral hydronephrosis: an explanation for the controversy about poor drainage. BJU Int. 2001;87(6):551–5. 11298056 10.1046/j.1464-410X.2001.00081.x

[16] Shokeir AA, Nijman RJ. Primary megaureter: current trends in diagnosis and treatment. BJU Int. 2000;86(7):861–8. 11069415 10.1046/j.1464-410x.2000.00922.x

[17] Blickman JG, Lebowitz RL. The coexistence of primary megaureter and reflux. AJR Am J Roentgenol. 1984;143(5):1053–7. 6333147 10.2214/ajr.143.5.1053

[18] Aaronson DS, Siddiqui SA, Reinberg Y, Baskin LS. Relative contraindication to endoscopic subureteral injection for vesicoureteral reflux: congenital refluxing megaureter with distal aperistaltic segment. Urology. 2008;71(4):616–9; discussion 619–620. 10.1016/j.urology.2007.11.086 18295311

[19] García-Aparicio L, Rodo J, Palazon P, Martín O, Blázquez-Gómez E, Manzanares A, et al. Acute and delayed vesicoureteral obstruction after endoscopic treatment of primary vesicoureteral reflux with dextranomer/hyaluronic acid copolymer: why and how to manage. J Pediatr Urol. 2013;9(4):493–7. 23507288 10.1016/j.jpurol.2013.02.007

[20] Chertin B, Mele E, Kocherov S, Zilber S, Gerocarni Nappo S, Capozza N. What are the predictive factors leading to ureteral obstruction following endoscopic correction of VUR in the pediatric population? J Pediatr Urol. 2018;14(6):538.e1–538.e7. 29885870 10.1016/j.jpurol.2018.04.021

[21] Friedmacher F, Colhoun E, Puri P. Endoscopic injection of dextranomer/hyaluronic acid as first line treatment in 851 consecutive children with high grade vesicoureteral reflux: Efficacy and long-term results. J Urol. 2018;200(3):650–5. 29551405 10.1016/j.juro.2018.03.074

[22] Castagnetti M, Cimador M, Sergio M, De Grazia E. Double-J stent insertion across vesicoureteral junction--is it a valuable initial approach in neonates and infants with severe primary nonrefluxing megaureter? Urology. 2006;68(4):870–5; discussion 875–876. 10.1016/j.urology.2006.05.052 17070371

[23] Carroll D, Chandran H, Joshi A, McCarthy LSL, Parashar K. Endoscopic placement of double-J ureteric stents in children as a treatment for primary obstructive megaureter. Urol Ann. 2010;2(3):114–8. 20981199 10.4103/0974-7796.68860 PMC2955226

[24] Farrugia MK, Steinbrecher HA, Malone PS. The utilization of stents in the management of primary obstructive megaureters requiring intervention before 1 year of age. J Pediatr Urol. 2011;7(2):198–202. 20494618 10.1016/j.jpurol.2010.04.015

[25] Awad K, Woodward MN, Shalaby MS. Long-term outcome of JJ stent insertion for primary obstructive megaureter in children. J Pediatr Urol. 2019;15(1):66.e1–66.e5. 30385050 10.1016/j.jpurol.2018.09.011

[26] Lee SD, Akbal C, Kaefer M. Refluxing ureteral reimplant as temporary treatment of obstructive megaureter in neonate and infant. J Urol. 2005 Apr;173(4):1357–60; discussion 1360. 10.1097/01.ju.0000152317.72166.df 15758801

[27] Kaefer M, Misseri R, Frank E, Rhee A, Lee SD. Refluxing ureteral reimplantation: a logical method for managing neonatal UVJ obstruction. J Pediatr Urol. 2014;10(5):824–30. 24850437 10.1016/j.jpurol.2014.01.027

[28] Alyami FA, Koyle MA, Bowlin PR, Gleason JM, Braga LH, Lorenzo AJ. Side-to-side refluxing nondismembered ureterocystotomy: a novel strategy to address obstructed megaureters in children. J Urol. 2017;198(5):1159–67. 28571679 10.1016/j.juro.2017.05.078

[29] Angulo JM, Arteaga R, Rodríguez Alarcón J, Calvo MJ. Role of retrograde endoscopic dilatation with balloon and derivation using double pig-tail catheter as an initial treatment for vesico-ureteral junction stenosis in children. Cirugia Pediatr Organo Of Soc Espanola Cirugia Pediatr. 1998;11(1):15–8. 9662865

[30] Angerri O, Caffaratti J, Garat JM, Villavicencio H. Primary obstructive megaureter: initial experience with endoscopic dilatation. J Endourol. 2007;21(9):999–1004. 17941775 10.1089/end.2006.0122

[31] Christman MS, Kasturi S, Lambert SM, Kovell RC, Casale P. Endoscopic management and the role of double stenting for primary obstructive megaureters. J Urol. 2012;187(3):1018–22. 22264463 10.1016/j.juro.2011.10.168

[32] García-Aparicio L, Rodo J, Krauel L, Palazon P, Martin O, Ribó JM. High pressure balloon dilation of the ureterovesical junction–first line approach to treat primary obstructive megaureter? J Urol. 2012;187(5):1834–8. 22425047 10.1016/j.juro.2011.12.098

[33] Torino G, Collura G, Mele E, Garganese MC, Capozza N. Severe primary obstructive megaureter in the first year of life: preliminary experience with endoscopic balloon dilation. J Endourol. 2012;26(4):325–9. 22050492 10.1089/end.2011.0399

[34] García-Aparicio L, Blázquez-Gómez E, Martin O, Palazón P, Manzanares A, García-Smith N, et al. Use of high-pressure balloon dilatation of the ureterovesical junction instead of ureteral reimplantation to treat primary obstructive megaureter: is it justified? J Pediatr Urol. 2013;9(6 Pt B):1229–33. 23796389 10.1016/j.jpurol.2013.05.019

[35] Romero RM, Angulo JM, Parente A, Rivas S, Tardáguila AR. Primary obstructive megaureter: The role of high pressure balloon dilation. J Endourol. 2014;28(5):517–23. 24400855 10.1089/end.2013.0210

[36] Bujons A, Saldaña L, Caffaratti J, Garat JM, Angerri O, Villavicencio H. Can endoscopic balloon dilation for primary obstructive megaureter be effective in a long-term follow-up? J Pediatr Urol. 2015;11(1):37.e1-6. 25748631 10.1016/j.jpurol.2014.09.005

[37] Capozza N, Torino G, Nappo S, Collura G, Mele E. Primary obstructive megaureter in infants: our experience with endoscopic balloon dilation and cutting balloon ureterotomy. J Endourol. 2015;29(1):1–5. 24646018 10.1089/end.2013.0665

[38] García-Aparicio L, Blázquez-Gómez E, de Haro I, Garcia-Smith N, Bejarano M, Martin O, et al. Postoperative vesicoureteral reflux after high-pressure balloon dilation of the ureterovesical junction in primary obstructive megaureter. Incidence, management and predisposing factors. World J Urol. 2015;33(12):2103–6. **The primary study on POM balloon dilation to systematically assess for postoperative VUR**. 25899625 10.1007/s00345-015-1565-9

[39] Kassite I, Braïk K, Morel B, Villemagne T, Szwarc C, Maakaroun Z, et al. High pressure balloon dilatation of the ureterovesical junction in primary obstructive megaureter: Infectious morbidity. Progres En Urol J Assoc Francaise Urol Soc Francaise Urol. 2017;27(10):507–12. 10.1016/j.purol.2017.07.005 28867581

[40] Casal Beloy I, Somoza Argibay I, García González M, García Novoa MA, Míguez Fortes LM, Dargallo CT. Endoscopic balloon dilatation in primary obstructive megaureter: Long-term results. J Pediatr Urol. 2018;14(2):167.e1–167.e5. 29398584 10.1016/j.jpurol.2017.10.016

[41] Kassite I, Renaux Petel M, Chaussy Y, Eyssartier E, Alzahrani K, Sczwarc C, et al. High pressure balloon dilatation of primary obstructive megaureter in children: a multicenter study. Front Pediatr. 2018;6:329. 30430104 10.3389/fped.2018.00329 PMC6220115

[42] Ortiz R, Parente A, Perez-Egido L, Burgos L, Angulo JM. Long-term outcomes in primary obstructive megaureter treated by endoscopic balloon dilation. Experience after 100 cases. Front Pediatr. 2018;6:275. **Largest study on POM balloon dilation in the literature, to date**. 30345263 10.3389/fped.2018.00275 PMC6182095

[43] Chiarenza SF, Bleve C, Zolpi E, Battaglino F, Fasoli L, Bucci V. Endoscopic balloon dilatation of primary obstructive megaureter: method standardization and predictive prognostic factors. Pediatr Medica E Chir Med Surg Pediatr. 2019;41(2):25–8. 10.4081/pmc.2019.219 31867939

[44] Torino G, Roberti A, Brandigi E, Turrà F, Fonzone A, Di Iorio G. High-pressure balloon dilatation for the treatment of primary obstructive megaureter: is it the first line of treatment in children and infants? Swiss Med Wkly. 2021;7(151):w20513. 10.4414/smw.2021.20513 34161596

[45] Faraj S, Loubersac T, Graveleau A, Alliot H, Camby C, Leclair MD. Postoperative JJ stent is not necessary after balloon high-pressure endoscopic dilatation of primary obstructive megaureter. J Pediatr Urol. 2022;18(3):369.e1–369.e7. 35562267 10.1016/j.jpurol.2022.03.028

[46] Contini G, Mele E, Capozza N, Castagnetti M. Endoscopic balloon dilatation for the treatment of primary obstructive megaureter <24 months of age: Does the size of the balloon influence results? J Pediatr Urol. 2022;S1477–5131(22):00536–8. 10.1016/j.jpurol.2022.11.021 36494270

[47] Boswell TC, Davis-Dao CA, Williamson SH, Chamberlin JD, Nguyen T, Chuang KW, et al. Endoscopic treatment of primary obstructive megaureter with high pressure balloon dilation in infants. J Pediatr Urol. 2023;S1477–5131(23):00407–12. 10.1016/j.jpurol.2023.09.007 37783596

[48] Kajbafzadeh AM, Payabvash S, Salmasi AH, Arshadi H, Hashemi SM, Arabian S, et al. Endoureterotomy for treatment of primary obstructive megaureter in children. J Endourol. 2007;21(7):743–9. 17705763 10.1089/end.2006.0330

[49] Smeulders N, Yankovic F, Chippington S, Cherian A. Primary obstructive megaureter: cutting balloon endo-ureterotomy. J Pediatr Urol. 2013;9(5):692.e1–2. 23759477 10.1016/j.jpurol.2013.04.010

[50] Shirazi M, Natami M, Hekmati P, Farsiani M. Result of endoureterotomy in the management of primary obstructive megaureter in the first year of life: preliminary report. J Endourol. 2014;28(1):79–83. 23937376 10.1089/end.2013.0098

[51] Doudt AD, Pusateri CR, Christman MS. Endoscopic management of primary obstructive megaureter: a systematic review. J Endourol. 2018;32(6):482–7. 29676162 10.1089/end.2017.0434

[52] Aiello G, Morlacco A, Bianco M, Soligo M, Meneghesso D, Vidal E, et al. Efficacy and safety of high-pressure balloon dilatation for primary obstructive megaureter in children: A systematic review. Front Urol [Internet]. 2022 [cited 2022 Nov 15];2. Available from: https://www.frontiersin.org/articles/10.3389/fruro.2022.1042689. **Thorough systematic review of POM balloon dilation**.

[53] Skott M, Gnech M, Hoen LA ’t, Kennedy U, Van Uitert A, Zachou A, et al. Endoscopic dilatation/incision of primary obstructive megaureter. A systematic review. On behalf of the EAU paediatric urology guidelines panel. J Pediatr Urol [Internet]. 2023 Sep 13 [cited 2023 Nov 26]; Available from: https://www.sciencedirect.com/science/article/pii/S1477513123004047. **Highest level systematic review of POM balloon dilation studies to date**. 10.1016/j.jpurol.2023.09.005 37758534

[54] Ripatti L, Viljamaa HR, Suihko A, Pakkasjärvi N. High-pressure balloon dilatation of primary obstructive megaureter in children: a systematic review. BMC Urol. 2023;23(1):30. **Thorough systematic review of POM balloon dilation**. 36869342 10.1186/s12894-023-01199-5 PMC9985206

[55] Babu R, Chandrasekharam VVS. A systematic review and meta-analysis comparing outcomes of laparoscopic extravesical versus trans vesicoscopic ureteric reimplantation. J Pediatr Urol. 2020;16(6):783–9. 33023851 10.1016/j.jpurol.2020.09.006

[56] Jayanthi VR. Vesicoscopic cross-trigonal ureteral reimplantation: High success rate for elimination of primary reflux. J Pediatr Urol. 2018;14(4):324.e1–324.e5. 29748123 10.1016/j.jpurol.2018.04.005

[57] Choi H, Park JY, Bae JH. Initial experiences of laparoscopic intravesical detrusorraphy using the Politano-Leadbetter technique. J Pediatr Urol. 2016;12(2):110.e1–7. 26750185 10.1016/j.jpurol.2015.07.014

[58] Chung KLY, Sihoe J, Liu K, Chao N, Hung J, Liu C, et al. Surgical outcome analysis of pneumovesicoscopic ureteral reimplantation and endoscopic dextranomer/hyaluronic acid injection for primary vesicoureteral reflux in children: a multicenter 12-year review. J Laparoendosc Adv Surg Tech A. 2018;28(3):348–53. 29271690 10.1089/lap.2017.0281

[59] Benaired AM, Zahaf H, Bourazi N. Pneumovesicoscopic Correction of Primary Vesicoureteral Reflux in Children: Our Initial Experience. J Endourol. 2021;35(12):1808–12. 34060919 10.1089/end.2020.1217

[60] Rudin YE, Marukhnenko DV, Galitskaya DA, Aliev JK, Lagutin GV, Vardak AB. Pneumovesicoscopic ureteral reimplantation with intravesical tailoring of obstructive megaureter in pediatric patient. J Pediatr Urol. 2022;18(2):224.e1–224.e8. 34991990 10.1016/j.jpurol.2021.12.004

[61] Kruppa C, Wilke A, Hörz C, Kosk T, Hörz T, Fitze G, et al. Vesicoscopic vs. open ureteral reimplantation according to cohen and leadbetter-politano for vesicoureteral reflux. J Clin Med. 2023;12(17):5686. 37685751 10.3390/jcm12175686 PMC10488379

[62] Nishi M, Eura R, Hayashi C, Gohbara A, Yamazaki Y. Vesicoscopic ureteral reimplantation with a modified Glenn-Anderson technique for vesicoureteral reflux. J Pediatr Urol. 2023;19(3):322.e1–322.e7. 36959038 10.1016/j.jpurol.2023.02.018

[63] Marchini GS, Hong YK, Minnillo BJ, Diamond DA, Houck CS, Meier PM, et al. Robotic assisted laparoscopic ureteral reimplantation in children: case matched comparative study with open surgical approach. J Urol. 2011;185(5):1870–5. 21421223 10.1016/j.juro.2010.12.069

[64] Smith RP, Oliver JL, Peters CA. Pediatric robotic extravesical ureteral reimplantation: comparison with open surgery. J Urol. 2011;185(5):1876–81. 21421231 10.1016/j.juro.2010.12.072

[65] Akhavan A, Avery D, Lendvay TS. Robot-assisted extravesical ureteral reimplantation: outcomes and conclusions from 78 ureters. J Pediatr Urol. 2014;10(5):864–8. 24642080 10.1016/j.jpurol.2014.01.028

[66] Dangle PP, Shah A, Gundeti MS. Robot-assisted laparoscopic ureteric reimplantation: extravesical technique. BJU Int. 2014;114(4):630–2. 24841534 10.1111/bju.12813

[67] Grimsby GM, Dwyer ME, Jacobs MA, Ost MC, Schneck FX, Cannon GM, et al. Multi-institutional review of outcomes of robot-assisted laparoscopic extravesical ureteral reimplantation. J Urol. 2015;193(5 Suppl):1791–5. 25301094 10.1016/j.juro.2014.07.128

[68] Boysen WR, Akhavan A, Ko J, Ellison JS, Lendvay TS, Huang J, et al. Prospective multicenter study on robot-assisted laparoscopic extravesical ureteral reimplantation (RALUR-EV): Outcomes and complications. J Pediatr Urol. 2018;14(3):262.e1–262.e6. 29503220 10.1016/j.jpurol.2018.01.020

[69] Hajiyev P, Sloan M, Fialkoff J, Gundeti MS. The LUAA gundeti technique for bilateral robotic ureteral reimplantation: lessons learned over a decade for optimal (resolution, urinary retention, and perioperative complications) trifecta outcomes. Eur Urol Open Sci. 2023;57:60–5. 37790798 10.1016/j.euros.2023.09.006 PMC10543781

[70] Deng T, Liu B, Luo L, Duan X, Cai C, Zhao Z, et al. Robot-assisted laparoscopic versus open ureteral reimplantation for pediatric vesicoureteral reflux: a systematic review and meta-analysis. World J Urol. 2018;36(5):819–28. 29374841 10.1007/s00345-018-2194-x

[71] Feng S, Yu Z, Yang Y, Bi Y, Luo J. Minimally invasive versus open ureteral reimplantation in children: a systematic review and meta-analysis. Eur J Pediatr Surg Off J Austrian Assoc Pediatr Surg Al Z Kinderchir. 2023. 10.1055/s-0043-1764321 36882103

[72] Esposito C, Castagnetti M, Autorino G, Coppola V, Cerulo M, Esposito G, et al. Robot-assisted laparoscopic extra-vesical ureteral reimplantation (ralur/revur) for pediatric vesicoureteral reflux: a systematic review of literature. Urology. 2021;1(156):e1–11. 10.1016/j.urology.2021.06.043 34324913

[73] Gnech M, ’t Hoen L, Zachou A, Bogaert G, Castagnetti M, O’Kelly F, et al. Update and Summary of the European Association of Urology/European Society of Paediatric Urology Paediatric Guidelines on Vesicoureteral Reflux in Children. Eur Urol. 2024;85(5):433–42. 10.1016/j.eururo.2023.12.005 38182493

[74] Sforza S, Marco BB, Haid B, Baydilli N, Donmez MI, Spinoit AF, et al. A multi-institutional European comparative study of open versus robotic-assisted laparoscopic ureteral reimplantation in children with high grade (IV-V) vesicoureteral reflux. J Pediatr Urol. 2023;S1477–5131(23):00506–15. **Recent high quality study of open versus robotic reimplantation for high grade VUR**. 10.1016/j.jpurol.2023.11.006 38000950

[75] Arlen AM, Broderick KM, Travers C, Smith EA, Elmore JM, Kirsch AJ. Outcomes of complex robot-assisted extravesical ureteral reimplantation in the pediatric population. J Pediatr Urol. 2016;12(3):169.e1–6. 26747012 10.1016/j.jpurol.2015.11.007

[76] Esposito C, Masieri L, Fourcade L, Ballouhey Q, Varlet F, Scalabre A, et al. Pediatric robot-assisted extravesical ureteral reimplantation (revur) in simple and complex ureter anatomy: Report of a multicenter experience. J Pediatr Urol. 2023;19(1):136.e1–136.e7. 36344364 10.1016/j.jpurol.2022.10.024

[77] Mittal S, Srinivasan A, Bowen D, Fischer KM, Shah J, Weiss DA, et al. Utilization of robot-assisted surgery for the treatment of primary obstructed megaureters in children. Urology. 2021;149:216–21. **Largest robotic reimplant series for POM**. 33129867 10.1016/j.urology.2020.10.015

[78] Bondarenko S. Laparoscopic extravesical transverse ureteral reimplantation in children with obstructive megaureter. J Pediatr Urol. 2013;9(4):437–41. 23491982 10.1016/j.jpurol.2013.01.001

[79] Neheman A, Shumaker A, Gal J, Haifler M, Kord E, Rappaport YH, et al. Robot-assisted laparoscopic extravesical cross-trigonal ureteral reimplantation with tailoring for primary obstructive megaureter. Urology. 2019;134:243–5. 31542465 10.1016/j.urology.2019.09.003

[80] Neheman A, Kord E, Koucherov S, Kafka I, Gaber J, Noh PH, et al. A novel surgical technique for obstructed megaureter: robot-assisted laparoscopic dismembered extravesical cross-trigonal ureteral reimplantation-short-term assessment. J Endourol. 2020;34(3):249–54. 31760787 10.1089/end.2019.0192

[81] Rappaport YH, Kord E, Noh PH, Koucherov S, Gaber J, Shumaker A, et al. Minimally invasive dismembered extravesical cross-trigonal ureteral reimplantation for obstructed megaureter: a multi-institutional study comparing robotic and laparoscopic approaches. Urology. 2021;1(149):211–5. 10.1016/j.urology.2020.10.018 33122054

[82] Li W, Dong H, Chen P, Su C, Wang C, Li Y, et al. Surgical management of vesicoureteral junction obstruction in children: a comparative study between transvesicoscopic cohen reimplantation and transumbilical laparoendoscopic single-site lich-gregoir techniques. J Endourol. 2022;36(8):1043–9. 35323047 10.1089/end.2021.0309

[83] Lopez M, Perez-Etchepare E, Bustangi N, Godik O, Juricic M, Varlet F, et al. Laparoscopic extravesical reimplantation in children with primary obstructive megaureter. J Laparoendosc Adv Surg Tech A. 2023;33(7):713–8. 32212997 10.1089/lap.2019.0396

[84] Li B, Lindgren BW, Liu DB, Gong EM. Robot-assisted laparoscopic megaureter tapering with ureteral reimplantation: Tips and tricks. J Pediatr Urol. 2017;13(6):637–8. 29079484 10.1016/j.jpurol.2017.09.013

[85] Zhu W, Zhou H, Cao H, Li P, Tao Y, Ma L, et al. Modified technique for robot-assisted laparoscopic infantile ureteral reimplantation for obstructive megaureter. J Pediatr Surg. 2022;57(12):1011–7. 35717252 10.1016/j.jpedsurg.2022.05.015

[86] Villanueva CA. Extracorporeal ureteral tailoring during HIDES laparoscopic robotic-assisted ureteral reimplantation for megaureter. J Pediatr Urol. 2015;11(6):362–3. 26455636 10.1016/j.jpurol.2015.08.006

[87] Sforza S, Cini C, Negri E, Bortot G, Di Maida F, Cito G, et al. Ureteral reimplantation for primary obstructive megaureter in pediatric patients: is it time for robot-assisted approach? J Laparoendosc Adv Surg Tech A. 2022;32(2):231–6. 34905408 10.1089/lap.2021.0246

[88] Bowen DK, Faasse MA, Liu DB, Gong EM, Lindgren BW, Johnson EK. Use of pediatric open, laparoscopic and robot-assisted laparoscopic ureteral reimplantation in the United States: 2000 to 2012. J Urol. 2016;196(1):207–12. 26880414 10.1016/j.juro.2016.02.065

[89] Garcia-Roig M, Travers C, McCracken CE, Kirsch AJ. National trends in the management of primary vesicoureteral reflux in children. J Urol. 2018;199(1):287–93. 28941917 10.1016/j.juro.2017.09.073

[90] Läckgren G, Cooper CS, Neveus T, Kirsch AJ. Management of vesicoureteral reflux: What have we learned over the last 20 years? Front Pediatr. 2021;31(9):650326. 10.3389/fped.2021.650326 PMC8044769 33869117

[91] Kalayeh K, Brian Fowlkes J, Schultz WW, Sack BS. The 5:1 rule overestimates the needed tunnel length during ureteral reimplantation. Neurourol Urodyn. 2021;40(1):85–94. 33017072 10.1002/nau.24526

[92] Babu R. ‘Mini reimplantation’ for the management of primary obstructed megaureter. J Pediatr Urol. 2016;12(2):103.e1–103.e4. 26490102 10.1016/j.jpurol.2015.08.017

[93] Ossandon F, Romanini MV, Torre M. A modified technique of ureteroplasty for megaureter in children. J Urol. 2005;174(4 Pt 1):1417–20. 16145453 10.1097/01.ju.0000173134.44999.a6

[94] Villanueva CA. “Mini” extravesical reimplant with “mini” tapering for infants younger than 6 months. J Pediatr Urol. 2019;15(3):256.e1–256.e5. 30777659 10.1016/j.jpurol.2019.01.004

[95] Friedman AA, Hanna MK. Split-cuff nipple technique of ureteral reimplantation in children with thick-walled bladders due to posterior urethral valves. Urology. 2015;85(1):199–204. 25444631 10.1016/j.urology.2014.09.023

[96] Villanueva CA, Nelson CA, Stolle C. Intravesical tunnel length to ureteral diameter ratio insufficiently explains ureterovesical junction competence: A parametric simulation study. J Pediatr Urol. 2015;11(3):144.e1–5. 25819375 10.1016/j.jpurol.2015.01.015

[97] Villanueva CA, Tong J, Nelson C, Gu L. Ureteral tunnel length versus ureteral orifice configuration in the determination of ureterovesical junction competence: A computer simulation model. J Pediatr Urol. 2018;14(3):258.e1–258.e6. 29496421 10.1016/j.jpurol.2018.01.009

[98] Babu R. Laparoscopic nipple invagination combined extravesical (NICE) reimplantation technique in the management of primary obstructed megaureter. J Pediatr Urol. 2023;19(4):425.e1–425.e6. 37019712 10.1016/j.jpurol.2023.03.023

[99] Liu W, Du G, Guo F, Ma R, Wu R. Modified ureteral orthotopic reimplantation method for managing infant primary obstructive megaureter: a preliminary study. Int Urol Nephrol. 2016;48(12):1937–41. 27590133 10.1007/s11255-016-1409-6

[100] Ben-Meir D, McMullin N, Kimber C, Gibikote S, Kongola K, Hutson JM. Reimplantation of obstructive megaureters with and without tailoring. J Pediatr Urol. 2006;2(3):178–81. 18947604 10.1016/j.jpurol.2005.05.010

[101] Farrugia MK, Whitaker RH. The search for the definition, etiology, and effective diagnosis of upper urinary tract obstruction: the Whitaker test then and now. J Pediatr Urol. 2019;15(1):18–26. 30602417 10.1016/j.jpurol.2018.11.011

[102] Świȩtoń D, Grzywińska M, Czarniak P, Gołȩbiewski A, Durawa A, Teodorczyk J, et al. The emerging role of MR urography in imaging megaureters in children. Front Pediatr. 2022;10:839128. 35402364 10.3389/fped.2022.839128 PMC8984115

